# Co-Microencapsulation of Anthocyanins from Cornelian Cherry Fruits and Lactic Acid Bacteria in Biopolymeric Matrices by Freeze-Drying: Evidences on Functional Properties and Applications in Food

**DOI:** 10.3390/polym12040906

**Published:** 2020-04-14

**Authors:** Iuliana Maria Enache, Aida Mihaela Vasile, Elena Enachi, Vasilica Barbu, Nicoleta Stănciuc, Camelia Vizireanu

**Affiliations:** Faculty of Food Science and Engineering, Dunărea de Jos University of Galati, Romania, 111, Domnească Street, 800201 Galati, Romania; enacheiulianamaria@gmail.com (I.M.E.); aida.vasile@ugal.ro (A.M.V.); elena.ionita@ugal.ro (E.E.); vasilica.barbu@ugal.ro (V.B.); nsava@ugal.ro (N.S.)

**Keywords:** *Cornus mas*, *Lactobacillus casei*, microencapsulation, anthocyanins, functionalization

## Abstract

*Cornus mas* was used in this study as a rich source of health-promoting bioactives. The cornelian cherries were used to extract the polyphenols and anthocyanins. The chromatographic profile of the *Cornus mas* fruit extract revealed the presence of several anthocyanins, mainly delphinidin, cyanidin and pelargonidin glycosides. The extract was co-microencapsulated with *Lactobacillus casei* ssp. *paracasei* in a unique combination of whey protein isolates, inulin and chitosan by freeze-drying, with an encapsulation efficiency of 89.16 ± 1.23% for anthocyanins and 80.33 ± 0.44% for lactic acid bacteria. The pink-red colored powder showed a total anthocyanins content of 19.86 ± 1.18 mg cyanidin-3-glucoside/g dry weight (DW), yielding an antioxidant activity of 54.43 ± 0.73 mMol Trolox/g DW. The viable cells were 9.39 × 10^9^ colony forming units (CFU)/g DW. The confocal microscopy analysis revealed the microencapsulated powder as a complex one, with several large formations containing smaller aggregates, consisting of the lactic acid bacteria cells, the cornelian cherries’ bioactive compounds and the biopolymers. The powder was tested for stability over 90 days, showing a decrease of 50% in anthocyanins and 37% in flavonoids content, with no significant changes in antioxidant activity and CFU. The powder showed a significant inhibitory effect against the α-amylase of 89.72 ± 1.35% and of 24.13 ± 0.01% for α-glucosidase. In vitro digestibility studies showed a significant release of anthocyanins in gastric juice, followed by a decrease in intestinal simulated conditions. The functional properties of the powder were tested by addition into a yogurt, highlighting a higher and more stable antioxidant activity at storage when compared to the control.

## 1. Introduction

Considering the poor health status of today’s people, determined by numerous biotic and abiotic factors, numerous studies have recently reported the desirability of utilizing plant sources as resources for biologically active compounds with an effective role in a superior promotion of the health status [1]. In particular, anthocyanins are responsible for the colors of many fruits and vegetables and have been widely recognized as powerful antioxidants. Additionally, these diet-based compounds have been confirmed to possess anti-inflammatory, anti-carcinogenic, anti-mutagenic and chemopreventive effects in various in vitro animal studies and clinical experiments [2,3]. Several studies reported that dietary polyphenolics, including anthocyanins from berries, displayed potent antidiabetic effects, thus representing a promising source of functional foods with antidiabetic properties [4,5].

Anthocyanins may interrupt, or reverse, the carcinogenesis process by acting on intracellular signaling molecules involved in the initiation and/or promotion of the progress of cancer, in a cell type- and dose-dependent manner [6]. Depending on their specific structures, anthocyanins affect different cellular signaling elements that are crucial for the regulation of cell proliferation, as explained by Jing et al. [7]. Cornelian cherry (*Cornus mas* L.) is found mainly as a rich source of anthocyanins, in Southeast Asia. The strong antioxidative effect is attributed to phenolic compounds including phenolic acids, flavonoids, tannins and anthocyanins [8]. Despite the protective activity of polyphenols, their inefficient delivery systems and poor bioavailability strongly limit their application in medicine and functional food [9]. However, anthocyanins are prone to degradation by processing and storage conditions, such as temperature, light, pH and oxygen [10]. The poor stability of anthocyanins under mild alkaline conditions such as those typical in the intestinal tract can also lower their bioavailability.

One of the most studied and used technologies for protecting bioactives from degradation is microencapsulation. The technique includes entrapping the active ingredients within wall materials to form microcapsules. For a controlled targeted site of delivery, such as the colon or small intestine, a crucial step for an increased microencapsulation efficiency and microcapsules properties is wall material selection. For food and pharmaceutical applications, natural biopolymers including starches, dairy proteins and natural gums are food compatible and safe for use as wall materials.

Probiotics are known for multiple health benefits, such as: control of intestinal infections and serum cholesterol level, improvement of the lactose utilization and immune system, anticarcinogenic effects, reduction of gastrointestinal pain and treatment of bacterial infections [11,12]. However, many probiotic bacteria are unable to survive under adverse temperature and acidity environments, and thus stabilization techniques should be applied. Studies aiming for the valorization of plants and lactic acid bacteria with probiotic and prebiotic potential as sources of innovative natural products and/or molecules for both traditional and modern medicine are re-emerging [13]. However, the use of combinations of biologically active compounds and viable cells, such as anthocyanins and lactic bacteria, in the same microencapsulated formula for the purpose of the cumulative exploitation of benefits, are not commonly reported. Furthermore, selected anthocyanins may act as prebiotics, since in vitro studies revealed the modulatory effect of cyaniding-3-*O*-glucoside on *Bifidobacteria* and *Lactobacillus*, as the anthocyanin was metabolized into several small molecules by bacteria [14]. 

Therefore, the aim of our study was to obtain a functional food ingredient by co-microencapsulation of the cornelian cherry fruit aqueous extract with *Lactobacillus casei* by freeze drying. The microencapsulation materials were selected as whey proteins isolate (WPI), inulin and chitosan. The resulted powder was characterized in terms of encapsulation efficiency, both for anthocyanins and lactic bacteria, phytochemicals profile (anthocyanins, polyphenols and flavonoids), color and antioxidant activity. The powder was tested for phytochemicals stability and cell viability after storage at 4 °C for 3 months in the dark. Confocal laser microscopy was used to study the structure and morphology of the powder. Furthermore, in vitro digestibility was applied to test the release of the anthocyanins from microcapsules in a simulated environment. Additionally, in order to test the potential antidiabetic effect of the co-microencapsulated powder, the inhibitory effect on enzymes associated with carbohydrate metabolism, such as α-glucosidase and α-amylase, was tested. The co-microencapsulated powder was further used for food functionalization, by adding it into a yogurt in different ratios. The stability of bioactives was tested during 21 days of storage at 4–6 °C. Our study brings new insights into the co-microencapsulation of anthocyanins and lactic acid bacteria for the use of both as a new ingredient for food functionalization, in terms of color, radical scavenging and the antidiabetic potential.

## 2. Materials and Methods

### 2.1. Materials

#### Chemicals and Reagents

The experiments carried out in this study were performed using: acetic acid, hydrochloric acid, aluminum chloride, chitosan, 2,2-Diphenyl-1-picrylhydrazyl (DPPH), ethanol, Folin-Ciocalteu reagent, gallic acid, inulin from chicory, methanol, pancreatin (Kreon), pepsin from gastric porcine, potassium acetate, Trizma hydrochloride, (6-Hydroxy-2,5,7,8-tetramethylchromane-2-carboxylic acid) Trolox purchased by Sigma Aldrich (Taufkirchen, Germany), sodium bicarbonate from Honeywell, Fluka (Selze, Germany) and whey protein isolate 894 (WPI) from Fonterra (Clandeboye, New Zealand). Lactobacillus casei ssp. paracasei (L. casei 431^®^) strain was purchased from Chr. Hansen (Hoersholm, Denmark). de Man, Rogosa and Sharpe agar (MRS agar), purchased from Merck (Germany), was used for the evaluation of the viability of the L. casei 431^®^. All other chemicals were of analytical or HPLC grade.

### 2.2. Methods

#### 2.2.1. Extract Preparation and Sample Processing

The fruits used in the present study were purchased from a local market in Galati country (Romania) in October 2017. In order to obtain the extract, 50 g of fruits were washed with distillate water and dried with a paper towel. After that, the stones were removed and the pulp was grinded in order to obtain a compact mixture. The extract was prepared using hot water extraction at 45 °C in a ratio of 1:2, followed by ultrasonication for 30 min at 40 ± 3 °C (MRC Scientific Instruments, Holon, Israel). The ultrasonic bath is equipped with a digital control system of sonication time, temperature and frequency. The extraction was performed at a constant frequency of 40 kHz, at a constant power of 100 W. Cold water was added to maintain a constant temperature (40 ± 3 °C) in the ultrasonic bath. The samples were collected and centrifuged at 6000× *g* for 10 min at 4 °C. Furthermore, the resulting supernatant was used for microencapsulation experiments.

#### 2.2.2. Co-microencapsulation of Anthocyanins and Lactic Bacteria

A volume of 100 mL resulting from centrifugation was used to dissolve WPI, chitosan and inulin, in a ratio of 2:1:1 (*w*:*w*:*w*). The solutions were allowed to mix on a magnetic stirrer until complete hydration at 650 rpm. After homogenization, the solutions were sterilized using a UV lamp and inoculated with 1 g of *L. casei 431^®^* lyophilized culture (with an initial CFU of 10^11^/g) and freeze-dried (CHRIST Alpha 1-4 LD plus, Osterode am Harz, Germany) at −42 °C under a pressure of 10 Pa for 48 h. Afterwards, the powder was collected and packed in metallized bags, and kept at 4 °C until further analysis.

#### 2.2.3. The Phytochemical Characterization of the Extract and Powder

The extract and powder were characterized in terms of the anthocyanins content by a pH differential method, the total polyphenols content (TPC) using the Folin-Ciocalteu method, expressed as mg gallic acid (GAE)/g DW and antioxidant activity, expressed as mg Trolox/g DW, as described by Vasile et al. [15]. For the phytochemicals analysis of the powder, 0.2 g were extracted as described at the total anthocyanins content (TAC) for the encapsulation efficiency evaluation. 

#### 2.2.4. HPLC Analysis of the Anthocyanins 

The chromatographic elution profile of the cornelian cherries’ anthocyanins extract was analysed with a Thermo Finnigan Surveyor HPLC system coupled with a Diode-Array Detector, controlled by the Xcalibur software (Finnigan Surveyor LC, Thermo Scientific, Waltham, MA, USA). Before the chromatographic analysis, the cornelian cherries extract was filtered through a special C18 Sep-Pack cartridge (Cartridge-Waters, Milford, MA, USA) in order to separate only the anthocyanins and through 0.22 μm syringe filters (Bio Basic Canada Inc., Toronto, ON, Canada). To achieve this particular analysis, a C18 Synergi 4u Fusion-RP 80A column (150 × 4.6 mm, 4 μm) was used, at a column temperature of 27 °C. The elution parameters were: the injection volume was 10 μL, at a flow rate of 1 mL/min. The mobile phase consisted of two phases: 100% HPLC grade methanol (A) and 10% formic acid in Milli Q water (B), whereas the elution was achieved under the following gradient conditions: 0–20 min, 9%–35% (A); 20–30 min, 35% (A); 30–40 min, 35%–50% (A); and 40–55 min, 50%–9% (A). The detection of the anthocyanins was performed at a 520 nm wavelength. The chromatographic separation and identification of the targeted anthocyanins was assessed based on their retention times and peak areas with respect to their corresponding standard compounds (Sigma Aldrich, Darmstadt, Germany; Extrasynthese, Lyon, France) and to the chromatographic data stipulated in the literature [16,17,18]. 

### 2.3. Encapsulation Efficiency

The encapsulation efficiency was estimated by evaluation of the surface anthocyanins content (SAC) and total anthocyanins content (TAC), as described by Enache et al. [19]. Briefly, 0.2 g of co-microencapsulated powder were dissolved in 5 mL of methanol:acetic acid:distillated water mixture, in a ratio of 25:4:21, homogenized and sonicated to destroy the microparticles, followed by measuring the anthocyanins content by the pH differential method. The results are expressed as mg of cyanidin-3-glucoside equivalents (C3G) per g of dry weight of powder (mg/g DW). To investigate SAC, the procedure involved the addition of a mixture of methanol:ethanol in a ratio of 1:1 to 0.2 g of powder, followed by the same procedure as for TAC, skipping the homogenization and sonication steps. 

The encapsulation efficiency was calculated as follows:

(1) $$EE\left(\%\right)=\frac{TAC-SAC}{TAC}\times 100$$

For the lactic bacteria encapsulation efficiency estimation, the procedure described by Colín-Cruz et al. [20] was used. The quantification of the viable bacteria was performed by pour plate technique. The percentage (%) of efficiency was determined according to the following equation:

(2) $$EE\;\left(\%\right)=\frac{N}{N_{0}}\times 100$$

where *N* is the number of viable cells (CFU/g) in the powder, and *N*_0_ is the number of viable cells in the solution before the freeze-drying process.

#### 2.3.1. Viability of Lactic Acid Bacteria

In order to determine the viability of the cell counting of *L. casei 431^®^*, 10-fold serial dilutions of the samples were performed using a sterile physiological serum (0.9 g NaCl%, *w/v*), by using the pour plate technique. The viable cell number was determined by estimating the number of colony-forming units (CFU) by cultivation on the MRS-agar plates (medium at pH 5.7) after 48 h of aerobic incubation at 37 °C. The counts were expressed as CFU/g DW (ISO 8261 IDF122:2001). 

#### 2.3.2. In vitro Digestibility of the Anthocyanins 

A static digestion model involving simulated gastric juice at pH 2.0 and intestinal juice at pH 7.7 was applied to test the in vitro digestibility profile of anthocyanins from co-microencapsulated powder, as described by Oancea et al. [21].

#### 2.3.3. Inhibitory Activity

The powder was dissolved in sodium phosphate buffer (0.1 M, pH 6.9) at a concentration of 1 mg/mL. The α-glucosidase and α-amylase inhibitory activity of the co-microencapsulated powder was measured as described by Costamagna et al. [22]. 

#### 2.3.4. Structure and Morphological Analysis of the Co-microencapsulated Powder

The structure and morphology of the microencapsulated powder obtained by co-microencapsulating the biologically active compounds from cornelian cherries and *L. casei* 431^®^ freeze-dried culture within the WPI, chitosan and inulin biopolymer matrix were observed and determined with LSM 710 Carl Zeiss Confocal Laser scanning microscope (Carl Zeiss, Oberkohen, Germany). The aforementioned system uses four types of laser, namely a diode laser (405 nm), Ar-laser (458, 488, 514 nm), DPSS laser (diode pumped solid state–561 nm) and HeNe laser (633 nm). The images were captured and rendered with the black edition of the ZEN 2012 SP1 software (Carl Zeiss, Oberkohen, Germany). The microencapsulated samples’ fluorescence was assessed both in their unlabeled (native) and labeled with the Red Congo (40 μM) fluorophore.

#### 2.3.5. Colorimetric Analysis

For the color analysis of the powder, a CR 410 Chroma Meter (Konica Minolta, Tokyo, Japan) colorimeter was used to appreciate the selected coordinates, respectively: L* (illumination, brightness, 0 black, 100 white), a* (positive value red, negative value green,) and b* (positive value yellow, negative value blue). 

#### 2.3.6. Stability over the Time

The powder was analyzed for the phytochemical profile and cell viability after 90 days of storage at 4 °C in order to evaluate the stability of the bioactives.

#### 2.3.7. Value Added Food Product

In order to test the efficiency of the co-microencapsulated powder in terms of the ability for functionalization, the powder was added into a yogurt in different aleatory ratios of 2% and 5%. The added value yogurt samples were tested for phytochemicals and antioxidant activity over 21 days of storage at 4–6 °C. In parallel, a control test was obtained, without the addition of powder.

#### 2.3.8. Statistical Analysis

Minitab 19 statistical software was used to analyze the resulted data [23]. All experiments were performed in triplicate. The data are expressed as mean values followed by the standard deviation. A paired *t* test was performed to determine whether the mean of the differences between the two paired samples was similar or not.

## 3. Results

### 3.1. Phytochemical Profile of the Extract

**Figure 1:**
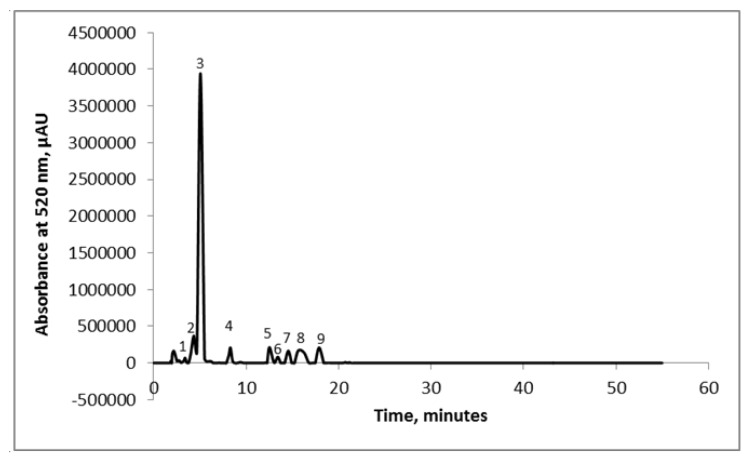
Chromatographic profile of the cornelian cherries’ extract: Peak 1—delphinidin-3-galactoside; Peak 2—cyanidin-3-glucoside; Peak 3—cyanidin-3-rutinoside; Peak 4—pelargonidin-3-glucoside; Peak 5—pelargonidin-3-rutinoside; and Peaks 6–9—unidentified compounds.

The global phytochemical profile of the extract showed TAC values of 1.23 ± 0.10 mg C3G/g DW, TPC of 2.66 ± 0.08 mg GAE/g DW, yielding an antioxidant activity of 15.89 ± 0.19 mMol Trolox/g DW. Hassanpour et al. [24] performed an extraction with methanol and hydrochloric acid and reported values for TPC ranging from 10.97 to 26.95 mg GAE/g FW. Popović et al. [25] suggested values for TAC ranging from 0.058 to 3.029 mg C3G/g DW for extraction performed with ethanol 80%. The chromatographic profile of the extract obtained from cornelian cherries’ extract displayed the presence of nine compounds, but only five were identified based on their corresponding standard and on the chromatographic data stipulated in the literature (Figure 1). 

The corresponding five compounds identified were: delphinidin-3-galactoside (1.03%), cyanidin-3-glucoside (5.42%), cyanidin-3-rutinoside (72.77%), pelargonidin-3-glucoside (3.05%) and pelargonidin-3-rutinoside (2.42%). The unidentified compounds represented less than 10% of the total anthocyanins content. The major two anthocyanins in the cornelian cherries’ extract were cyanidin-3-rutinoside with a content of 241.21 mg/100 g DW and cyanidin-3-glucoside (5.42%) with a content of 22.62 mg/100 g DW. The delphinidin-3-galactoside, pelargonidin-3-glucoside and pelargonidin-3-rutinoside contents were 4.31 mg/100 g DW, 12.73 mg/100 g DW and 9.12 mg/100 g DW, respectively. Dumitraşcu et al. [18] analyzed the anthocyanins’ content of cornelian cherries and revealed the presence of six compounds, out of which the highest content was registered for cyanidin-3-rutinoside. Nonetheless, Kucharska et al. [26] assessed the anthocyanins from 26 different cultivars of cornelian cherries and reported the existence of five major anthocyanins: delphinidin, cyanidin, and pelargonidin glycosides. 

### 3.2. Encapsulation Efficiency of Anthocyanins and Lactic Acid Bacteria

The encapsulation efficiency obtained in this study for anthocyanins was 89.16 ± 1.23% and 80.33 ± 0.44% for lactic acid bacteria. Oancea et al. [21] encapsulated anthocyanins from sour cherry skins in WPI and reported an encapsulation efficiency of 70.30 ± 2.20%, whereas Tao et al. [27] suggested that the encapsulation efficiency of blueberry anthocyanins within different ratios of WPI, gum acacia, maltodextrin and β-cyclodextrin was 82%. In order to check the stability of the bioactives and lactic acid bacteria from the powder, the encapsulation efficiency was measured after three months of storage in the dark at 4 °C. The encapsulation efficiency was 87.00 ± 1.56% for anthocyanins and 74.79 ± 0.71% for lactic acid bacteria. Fang et al. [28] suggested that after one year of storage the encapsulated anthocyanins of mulberry marc and wall materials (polyethylene glycol and chitosan) remained unchanged. Coelho-Rocha et al. [29] observed a decrease in the number of CFU (from 2.8 × 10^14^ CFU to 1.6 × 10^14^ CFU), this being about a 60% efficiency, in comparison with non-encapsulated ones. These authors observed that both strains (encapsulated and non-encapsulated) lost viability during five days of refrigeration. 

### 3.3. Global Phytochemical Profile of the Co-microencapsulated Powder

The extract showed a TAC content of 19.86 ± 1.18 mg C3G/g DW and TPC of 7.88 ± 0.22 mg GAE/g DW, yielding an antioxidant activity of 54.43 ± 0.73 mg Trolox/g DW. The powder showed an initial CFU of 9.39 × 10^9^/g DW. Vasile et al. [15] co-microencapsulated anthocyanins from black beans and *Lactobacillus casei* in whey proteins isolate, inulin and chitosan by freeze-drying*,* suggesting a phytochemicals content of TAC 1.65 ± 0.13 mg C3G/g DW, polyphenols of 21.64 ± 0.98 mg GAE/g DW and an antioxidant activity of 157.22 ± 4.13 mMol.

### 3.4. Structural and Morphological Analysis of the Powder

**Figure 2:**
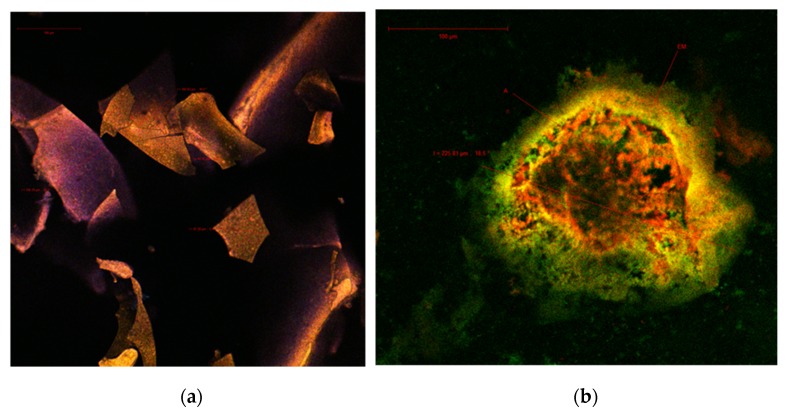
Confocal laser scanning microscopy images of (**a**) the native microencapsulated cornelian cherries powder and of (**b**) the fluorophore dyed microencapsulated cornelian cherries powder.

In order to determine the structure and morphological particularities of the co-microencapsulated anthocyanins and lactic bacteria in the biopolymeric matrix, the obtained powder was analyzed by confocal microscopy. The abundance of plant pigments with antioxidant properties in cornelian cherries has, along with their autofluorescence, been previously detailed by several other studies [18,30]. It is well known that phenolic compounds have a maximum absorption at 280 nm, carotenoids between 439 and 451 nm, and flavonoids and anthocyanins in the 520–580 nm range [31]. The point-by-point laser scanning of the native sample revealed several polygonal, irregular, large solziform formations, with sides ranging from 65.47 to 105.79 µm (Figure 2a), with an emission predominantly in the blue and yellow fields. The emission spectra of these biocomposites may overlap over a large domain, due to the unique ratio of these compounds in the vegetal tissue analyzed according to the variety or the harvest time. After the fluorescence marking, large spherosomes with a diameter of 225.81 μm (Figure 2b) were clearly observed, in which the anthocyanins (A - in yellow) and carotenoids (in red) were captured within the biopolymers’ wall, inulin, chitosan and WPI that formed the encapsulating matrix (EM - in green).

Due to the complex composition of the cornelian cherries’ microencapsulated powder, the confocal analysis did not allow for the visualization of the lactic bacteria that were encapsulated together with the antioxidant extract. 

### 3.5. In Vitro Digestibility of The Anthocyanins From The Powder

**Figure 3:**
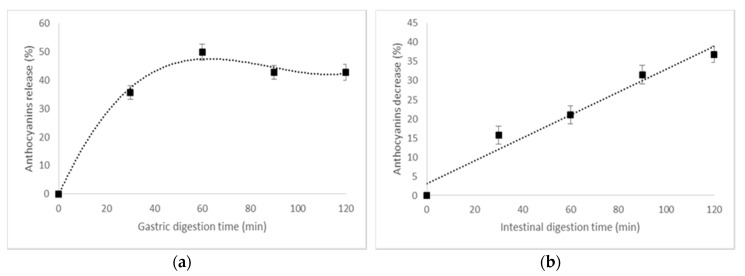
The in vitro digestibility pattern of anthocyanins in (**a**) a simulated gastric environment and in (**b**) intestinal juice.

The stability of anthocyanins in gastrointestinal conditions should be investigated to predict their bioavailability after consumption, since it has been reported that about 1% of the anthocyanins individuals consume are present in plasma [32]. For example, Vitaglione et al. [33] reported that protocatechuic acid is a major metabolite of cyanidin-3-*O*-glucoside in humans, which comprises 73% of all anthocyanins ingested by humans. In our study, the in vitro digestibility of the co-microencapsulated powder was evaluated in a static digestion system, involving testing the anthocyanins’ patterns in simulated gastric and intestinal juices. The release pattern of the anthocyanins from the co-microencapsulated powder is given in Figure 3. A significant release of anthocyanins was observed in the gastric phase, with a maximum of 50% after 60 min of digestion (Figure 3a). The anthocyanins decreased significantly in intestinal simulated juice (Figure 3b), with a maximum of approximatively 37% after 120 min of digestion.

González et al. [34] also reported that the anthocyanins from an encapsulated mix form by olive leaves’ extract in maltodextrin and inulin were partially degraded in gastric and intestinal conditions. These findings indicate that the instability of anthocyanins during gastrointestinal digestion should be considered when estimating the bioaccessibility and bioavailability of anthocyanins from co-microencapsulated powders. Further studies about the in vivo digestion of anthocyanins in different food matrices are needed to determine the actual health effects of anthocyanins when consumed.

### 3.6. In Vitro Antidiabetic Effects

The effective management of diabetes mellitus, especially the non-insulin-dependent Type II, comes in a context where it become epidemic in adults and children, leading to serious conditions, such as kidney failure, heart attack, blindness and lower limb amputation [35]. Secondly, the administration of synthetic enzyme inhibitors such as acarbose, sulfonylureas, biguanides, glinides, metformin and orlistat imply multiple side effects [36]. Therefore, it is necessary to develop new formulas for natural inhibitors of the starch digestive enzymes, such as α-amylase and α-glucosidase, thus preventing the excessive rise of the blood glucose level.

In this study, the co-microencapsulated powder was tested as a possible inhibitory formula for an antidiabetic effect at a concentration of 1 mg/mL. The powder exhibited a lower inhibitory effect against α-glucosidase of 24.13 ± 0.01% when compared with α-amylase, with an inhibitory effect of 89.72 ± 1.35%. Therefore, the co-microencapsulated powder was more effective against α-amylase and less effective on α-glucosidase. Costamagna et al. [22] reported that the simultaneous inhibition of both enzymes would result in abnormal bacterial fermentation in the colon due to the presence of undigested carbohydrates. Other plants, many of them used traditionally to control diabetes or hyperglycaemia, were reported to exert a strong inhibition of α-glucosidase and a moderate or negligible effect on the α-amylase activity [37].

### 3.7. Stability over Time of the Bioactive Compounds and Lactic Acid Bacteria

The stability of bioactive compounds and lactic acid bacteria were checked after three months of storage at 4 °C in the dark (Table 1). From Table 1, one can observe a slightly release of anthocyanins from microcapsules, with an increase of approximatively 4% and a decrease in TPC of aproximatively 41% in TPC, with no significant changes in the antioxidant activity. 

The obtained study is in good agreement with Moser et al. [38], suggesting that grape juice encapsulated with maltodextrin and soy or whey protein by spray drying had a high stability over the 150 days. Sultana et al. [39] and Vidhyalakshmi et al. [40] studied the effects of encapsulation in alginate–starch on the survival of *Lactobacillus acidophilus* and *Bifidobacterium spp*. in yogurt over a period of 60 days. These authors demonstrated that the survival of encapsulated cultures of *L. acidophilus* and *Bifidobacterium spp.* showed a decline in a viable count of about 0.5 log CFU/g DW over a period of 60 days, when compared with a 1 log CFU/g DW decrease for free cells. The co-microencapsulated powder showed a decrease in viable cells of *L. casei* 431*^®^* after 90 days with only 0.47 log CFU/g DW.

### 3.8. Color Intensity Analysis 

The data from the colorimetric analysis of the powder are shown in Table 2. 

Parameter a* may be correlated with the anthocyanins content in the powder, thus describing the tendency of the powder toward red, which can be associated to anthocyanidins released from proanthocyanidins [41] and the formation of anthocyanin-derived pigments that stabilize the red-coloured flavylium [38]. The L* parameter shows that powder encapsulated in whey proteins, chitosan and inulin has a tendency to light, since the encapsulation materials are white flourish materials, and it is natural that the colour of the powder is slightly lighter.

### 3.9. Testing the Co-microencapsulated Powder into Yogurt

In order to test the capacity of powder for food functionalization, different aleatory ratios of 2% (S1) and 5% (S2) were added into a yogurt. The products were tested for phytochemicals’ stability over 21 days at 4–6 °C. After 21 days, in S1 a threefold increase in the TAC content was observed, whereas a slow decrease in TFC with approximatively 7% was found, leading to a 14% decrease in the antioxidant activity. In S2, a 1.5-fold increase in TAC was found, suggesting a release in anthocyanins from microparticles, whereas the TPC was constant and the TFC decreased slightly at approximatively 12%, with no significant variations in the antioxidant activity. However, besides the significant bioactives content, both S1 and S2 showed an increased antioxidant activity when compared with the control. Thus, it was possible to test the value-added products, which exploited the content of the bioactive compounds of cornelian cherry and lactic bacteria, in a stable, co-microencapsulated state, from the perspective of developing stable, health-promoting ingredients, with multiple functions for food application.

## 4. Conclusions

The great opportunity of using plants as natural sources of biologically active compounds was exploited in our study, which aimed to extract polyphenols from cornelian cherry and to co-microencapsulate with lactic acid bacteria, from the perspective of obtaining ingredients with multiple functionalities, for nutraceutical and/or for food applications. The chromatographic profile of the cornelian cherries’ extract indicated that the high content of anthocyanins was attributed mainly to cyanidin-3-rutinoside and cyanidin-3-glucoside, which represented more than 78% of the total anthocyanins content. The extract and lactic acid bacteria were microencapsulated in whey proteins isolate, inulin and chitosan, with an encapsulation efficiency of almost 90% for anthocyanins and 80% for bacteria. The freeze-drying technique allowed us to obtain a pink-red powder, with a significant content of bioactives and viable cells of 9.39·× 10^9^ CFU/g DW. The morphology and structure’s analysis of the encapsulated powder displayed the presence of the bioactive compounds from cornelian cherries within some large formations with different dimensions. Significantly, the powder showed remarkable antioxidant and antidiabetic activities, with a higher inhibitory effect against α-amylase and a lower one for α-glucosidase. A storage stability test during 90 days at 4 C° showed a high stability of anthocyanins and lactic acid bacteria, preserving the antioxidant activity. The in vitro digestibility pattern highlighted a significant release of anthocyanins in gastric simulated conditions, and a decrease in intestinal simulated juice of approximatively 37% after 120 min of digestion. The powder was added in random concentrations of 2% and 5% into yogurt, and the stability of phytochemicals and antioxidant activity was tested at storage for 21 days at 4–6 °C. During storage, a slight decrease was observed in the antioxidant activity for the variant with a 2% powder addition, whereas for the variant with 5% no significant changes were found. The added-value was demonstrated by the fact that both the color and biologically active compounds content, in addition to the antioxidant activity, were significantly higher when compared to the control sample.

Our study demonstrates the potential for developing multi-functional ingredients, provided both by bioactives from plant sources and lactic acid bacteria, for applications as nutraceuticals or in food products. Additional studies are needed to demonstrate the efficiency of these powders for related health effects, such as hypocholesterolemic, anti-cancer, anti-inflammatory activities, etc.

## Figures and Tables

**Table 1:** Storage stability of co-microencapsulated bioactives.

Bioactives	Time 0	90 Days (3 Months)
Antioxidant activity (mMol Trolox/g DW)	54.43 ± 0.73 ^b^	59.86 ± 0.63 ^a^
Total polyphenolic content (mg GAE/g DW)	7.88 ± 0.22 ^a^	4.67 ± 0.37 ^b^
Total anthocyanins content (mg C3G/g DW)	19.86 ± 1.18 ^a^	20.51 ± 1.01 ^a^
Lactic acid bacteria (CFU/g DW)	9.39 × 10^9^	4.41 × 10^9^

The mean values that, for the same bioactive, do not share the same superscript letter (a, b) are statistically different at *p* < 0.01 based on the paired t test.

**Table 2:** CIELAB parameters of the co-microencapsulated powder.

L*	a*	b*
53.72 ± 0.82	20.22 ± 0.32	0.04 ± 0.03
